# Right ventricular dysfunction: a key predictor of post-intubation hypotension in the emergency department

**DOI:** 10.1186/s12245-025-00987-0

**Published:** 2025-09-30

**Authors:** Pranav Prakash, Nisarg S, Jayaraj Mymbilly Balakrishnan, Sai Deepak Alli, Ravi G. S, Siddhi Rajeev Naik

**Affiliations:** 1 Department of Emergency Medicine, Kasturba Medical College, Manipal, Manipal Academy of Higher Education, Manipal, Karnataka 576104 India; 2 Department of Biostatistics, National Institute of Mental Health and Neurosciences, Bengaluru, Karnataka 560029 India

**Keywords:** Post-intubation hypotension, TAPSE, Right ventricular dysfunction, POCUS, Shock index, Emergency airway management, Hemodynamic instability

## Abstract

**Background:**

Post-intubation hypotension (PIh) is a frequent complication following drug-assisted intubation, leading to increased morbidity, mortality, and healthcare costs. Pre-intubation hemodynamic factors, acid-base imbalances, and existing comorbidities, particularly right ventricular dysfunction, which is a lesser-known variable, have emerged as a critical predictor of PIh. Since RV systolic motion is predominantly longitudinal, TAPSE on POCUS provides a time-sensitive surrogate of RV function for high risk patients in the Emergency department.

**Methods:**

Aim: The study aimed to list hemodynamic predictors and their correlation with easily executable POCUS and point-of-care echocardiography variables, which can impact emergency decision-making and optimal management in PIh.

This prospective observational study was conducted in the Department of Emergency Medicine at Kasturba Medical College, Manipal, Manipal Academy of Higher Education, Manipal, Karnataka, India. 172 patients aged ≥ 18 years undergoing drug-assisted intubation were observed. Baseline demographics, clinical parameters, hemodynamic indices, and pre-intubation echocardiographic values (TAPSE, EPSS) were recorded. Shock index and modified shock index were calculated. Point-of-care ultrasound (POCUS) assessed left and right ventricular function. PIh within 30 min was defined as SBP $\:\le\:$ 90mmHg, ≥ 20% fall in SBP, MAP < 65mmHg or new vasopressor initiation. Associations were tested with logistic regression. TAPSE discrimination was obtained with ROC analysis and Youden’s Index.

**Results:**

Of the 172 patients, 71 (41.2%) developed PIh. Patients with obstructive lung disease (59.3%, *p* = 0.039) and sepsis (66.7%) were significantly more likely to experience PIh. TAPSE values were significantly lower in those with PIh (17.66 ± 2.45 mm vs. 18.54 ± 2.15 mm, *p* = 0.014). The multivariate logistic regression revealed TAPSE as an independent predictor of PIh (OR = 0.81, 95% CI = 0.69–0.95, *p* = 0.009). ROC analysis of TAPSE showed moderate predictive power (AUC 0.584, 95% CI 0.497–0.672), with a cut-off of 17.6 mm (sensitivity 85.1% and specificity 29.6%).

**Conclusion:**

TAPSE measured pre-intubation emerged as a reliable predictor of post-intubation hypotension. Incorporating a rapid assessment of right ventricular function using POCUS into the airway management algorithm provides valuable insights in identifying patients at higher risk of PIh.

**Supplementary Information:**

The online version contains supplementary material available at 10.1186/s12245-025-00987-0.

## Background

Timely airway management is one of the core elements of emergency medical care. The administration of an induction agent and a neuromuscular blocker during a drug-assisted intubation has significant implications for a patient’s peri- and post-intubation physiology in acute settings [1]. 

Hypotension is a commonly observed adverse phenomenon that incurs significant resource allocation and stress in the care environment in the immediate post-intubation period [2]. 

Post-intubation hypotension (PIh) is often associated with the need for immediate resuscitation, vasopressor requirements, prolonged ICU stay, and increased morbidity and mortality in patients, leading to increased healthcare costs.

Hence, prediction and pre-emptive care measures to avoid a significant fall in blood pressure and resultant end-organ damage are of primary importance. However, many risk factors are not fully delineated in the causation of PIh.

The emerging concept of the physiologically difficult airway deals with patients at an increased risk of hemodynamic instability or poor outcomes during intubation due to underlying physiological derangements such as hypoxemia, hypotension, severe metabolic acidosis, or right ventricular dysfunction. While the former four issues are well understood, the implications of right ventricular dysfunction are yet to be fully delineated and established.

The embryological origin of the right ventricle, with its muscle fibre arrangement, makes it more optimised for compliance and volume, and not pressure, behaving distinctly from the left ventricle [3]. Since the right ventricular contraction is predominantly by longitudinal fibres, Tricuspid Annular Plane Systolic Excursion (TAPSE) indicates the pulling of the tricuspid annulus towards the apex during systole.

The right ventricle is vulnerable to acute hemodynamic alterations that are commonly encountered during intubation, such as reduced preload, increased afterload and hence a decreased coronary perfusion pressure. This critical vulnerability shows us why patients with pre-existing RV dysfunction, objectively identified using point-of-care echocardiographic parameters, are susceptible to hemodynamic deterioration that manifests as post-intubation hypotension [4]. 

As patients are switched to mechanical ventilation, the positive airway pressure reduces venous return into the right atrium—lowering preload—while simultaneously elevating pulmonary pressures and thereby increasing right ventricular afterload [5]. 

This underscores that a point-of-care echocardiogram would become an invaluable tool for assessing critical hemodynamic states and functional cardiac reserves and their real-time stabilisation [6, 7]. 

However, there is a paucity of literature respective to the prediction of risk factors contributing to post-intubation hypotension using POCUS and echocardiography.

The study aimed to list hemodynamic predictors and their correlation with easily executable POCUS and point-of-care echocardiography variables, which can impact emergency decision-making and optimal management in PIh.

## Methods

We conducted a prospective observational study that evaluated patients 18 or older who underwent emergent drug-assisted endotracheal intubation in the Department of Emergency Medicine, Kasturba Medical College, Manipal, Manipal Academy of Higher Education, Manipal.

The study was conducted after the Institutional Ethics Committee approval (IEC2: 89/2023) and registration with the Clinical Trials Registry, India (CTRI/2023/05/052511).

Our study comprised 172 participants as per the calculated sample size [5]. The inclusion criteria encompassed all patients over 18 years of age presenting to the Emergency Medicine department who underwent drug-assisted endotracheal intubation, while we excluded non-consenting patients or kin, peri-arrest or established cardiac arrest, patients undergoing resuscitation, or those requiring reintubation.

Written informed consent was obtained from the next of kin or an appointed representative.

### Baseline medications & anthropometrics

As part of our enrolment proforma, we recorded each patient’s comorbidities, as well as approximated weight (from electronic medical records) and height.

#### Pharmacologic protocol for drug-assisted intubation

Our study was an observational study with non-interference, where all study subjects underwent a drug-assisted intubation technique. An induction agent and neuromuscular agent whose dose and method of intubation were based on the treating physician’s judgment of the patient’s hemodynamic status, comorbidity and existing medications. The details (agent and dose) were recorded on our enrolment proforma and subsequently extracted for analysis.

We recorded comorbidities and detailed pharmacological history of the patient at enrolment.

The treating team assessed, monitored, and stabilized the patients and performed a point-of-care echocardiography pre-intubation to measure bi-ventricular function and repeated post-intubation after half an hour or during hypotension. The hemodynamic parameters included monitoring of Shock Index, Modified Shock Index, TAPSE and EPSS values.

### Measurement of point-of-care echocardiographic parameters

**Fig. 1:**
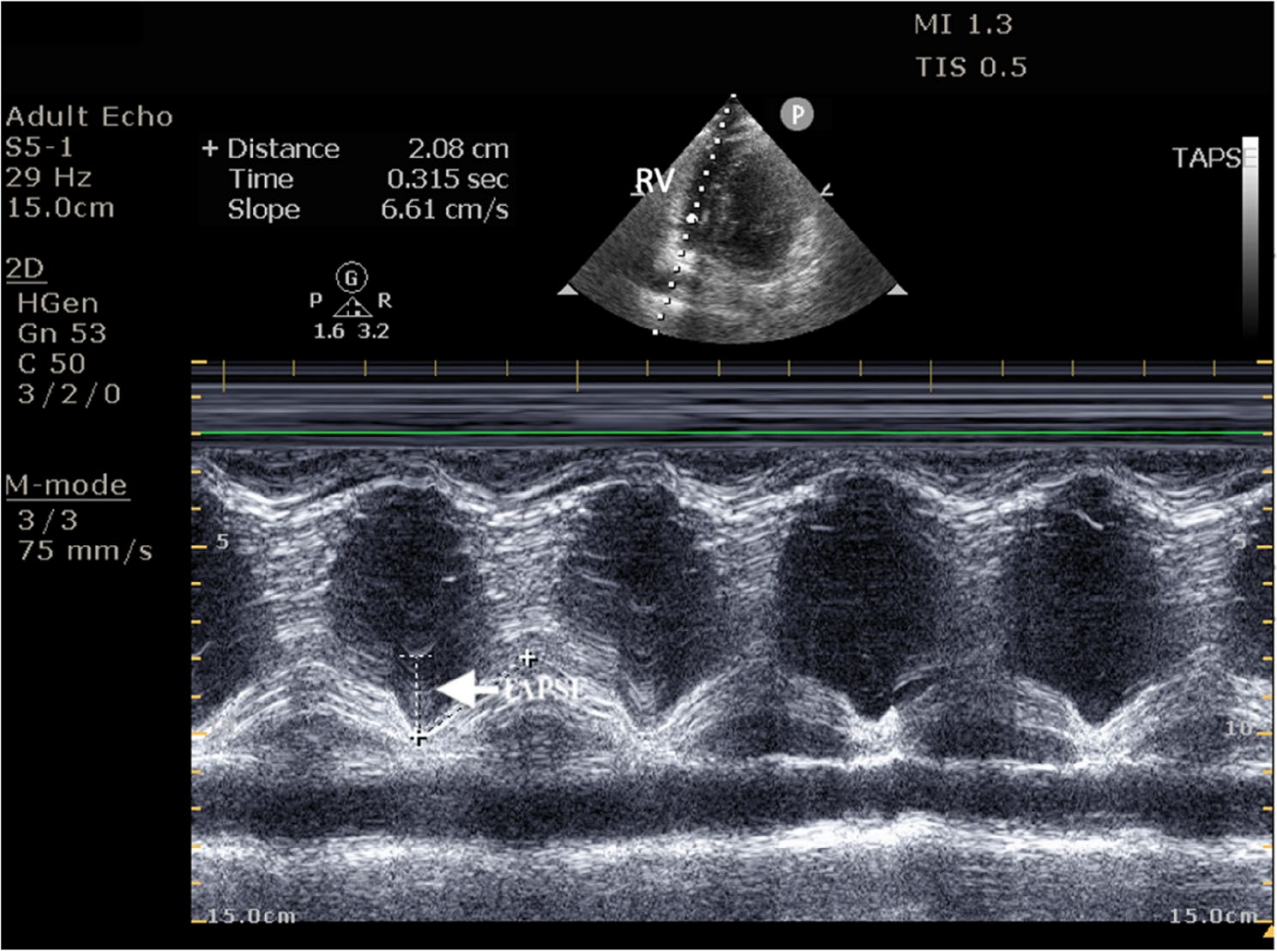
Echocardiographic measurement of TAPSE. Shows the measurement TAPSE (white arrow) depicted to be 20.8 mm. TAPSE = Tricuspid Annular Plane Systolic Excursion, RV = Right Ventricle

**Fig. 2:**
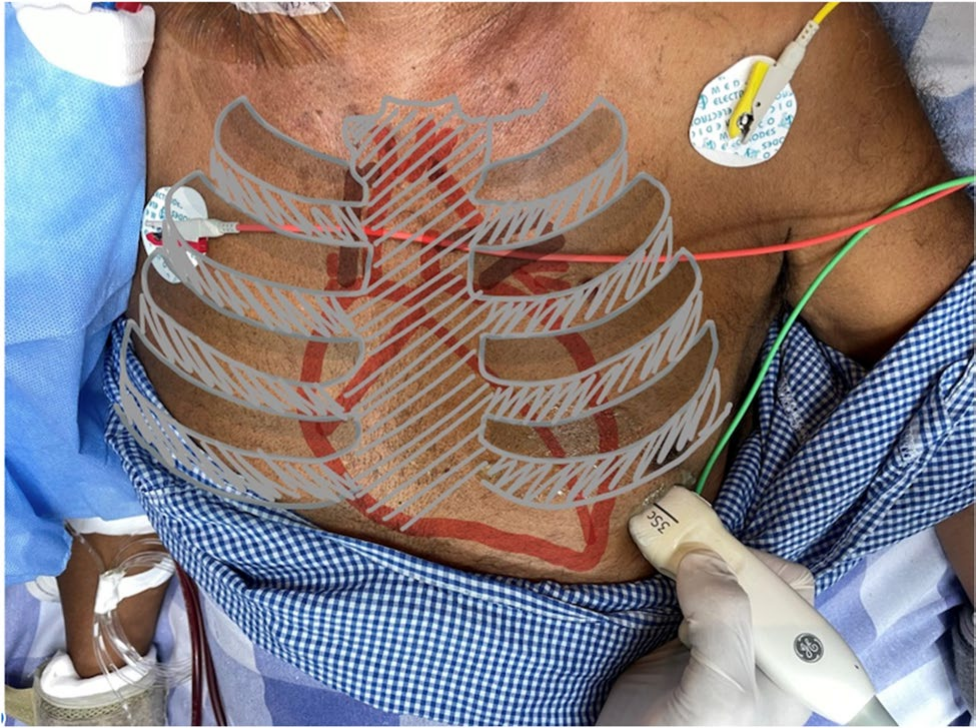
Probe placement for Apical 4 chamber view. Probe placed at the apex to obtain the 4- chamber view for TAPSE measurement

Right ventricular systolic function was quantified by measuring TAPSE in the Apical four-chamber view using M-mode, with the cursor placed at the lateral tricuspid annulus, as shown in Figs. 1 and 2.

**Fig. 3:**
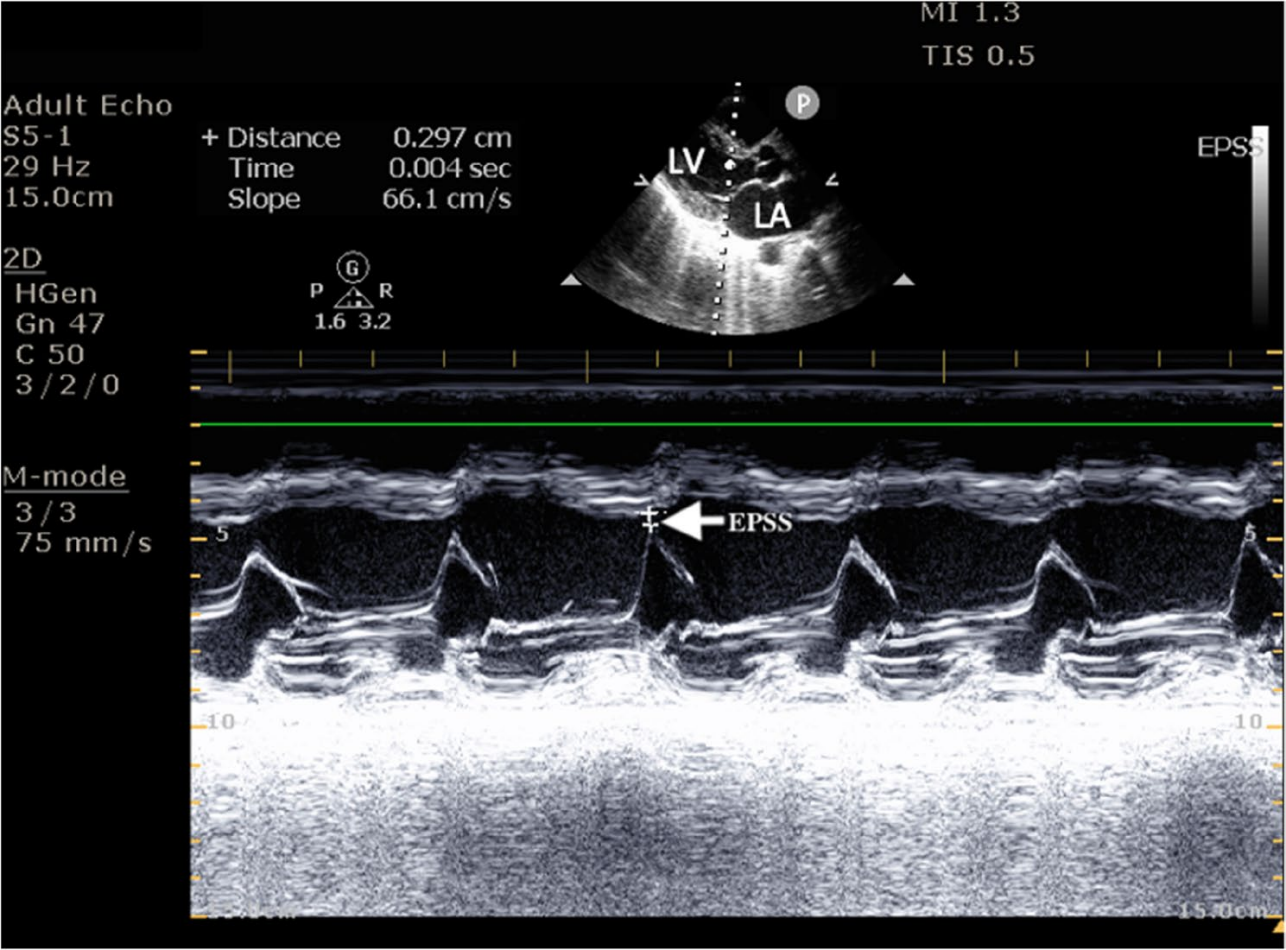
Echocardiographic Measurement of EPSS. Shows the echocardiographic view of EPSS measurement- 2.9 mm. EPSS = End Point Septal Separation, LV = Left Ventricle, LA- Left Atrium

**Fig. 4:**
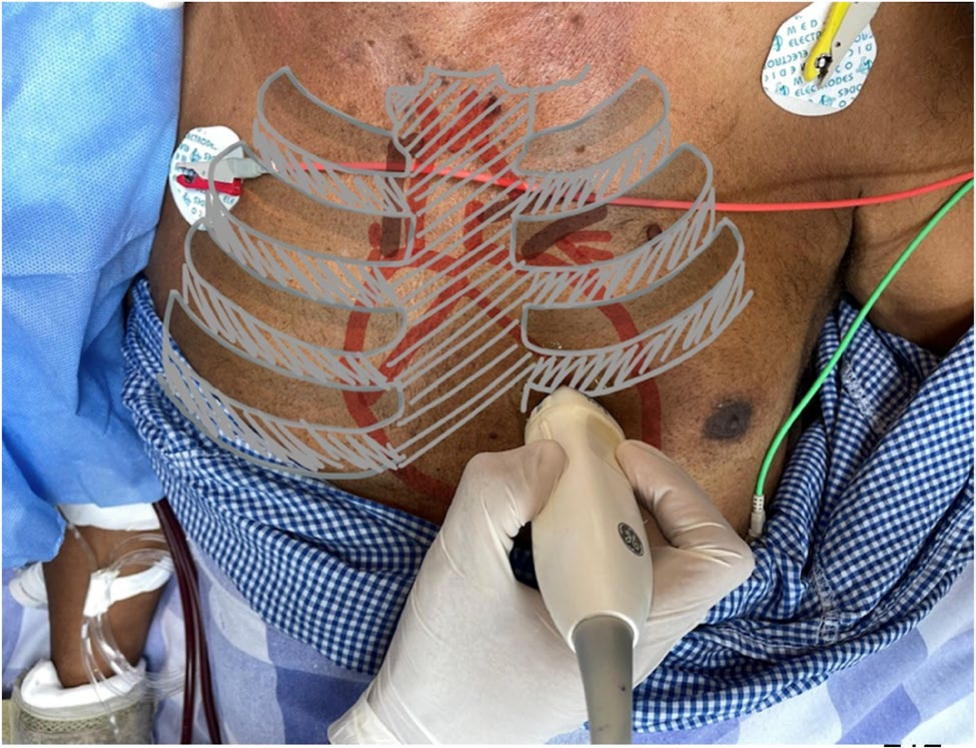
Probe placement for parasternal long axis view. Depicts prove placement in the parasternal position to obtain the PLAX view for EPSS Measurement. PLAX = Parasternal Long Axis

Left ventricular systolic function was quantified by measuring EPSS in parasternal long axis view and a horizontally oriented LV with the gate placed at the mitral valve tip in M mode, as shown in Figs. 3 and 4.

All operators completed a standardised training module to ensure reproducibility and reduce inter-operator variability. The module ensured that the investigator completed at least 25 supervised echocardiographic measurements with particular focus on TAPSE and EPSS, under the guidance of an expert echocardiographer.

### Definition of post-intubation hypotension

We defined PIh as the development of any of the following within 30 min of intubation -.

- A SBP of 90 mmHg OR
- A reduction from baseline SBP 20% OR
- Reduction in MAP of 65 mmHg OR
- Initiation of vasopressors

Measured either invasively through an arterial line or a non-invasive blood pressure cuff.

A reduction from baseline SBP of 20% or an increase in vasopressor requirement was considered post-intubation hypotension for a subset of patients who were hypotensive before intubation [8–10]. 

The data were analyzed using IBM-SPSS software (version 28). We also presented continuous or ordinal variables as mean ± standard deviation or median with interquartile range (IQR). Descriptive statistics were reported as frequencies and percentages for categorical variables. We examined bivariate associations between PIh and clinical variables using Chi-square or Fisher’s exact tests for categorical variables, as appropriate, and either the t-test with independent samples or the Mann-Whitney U test if normality was not met for continuous variables. Odds ratios with 95% confidence intervals were also reported for these comparisons. When data were sparse for certain subgroups, we utilized descriptive statistics. We performed all statistical tests at *p* < 0.05 significance threshold. Multivariable logistic regression was conducted based on the results of bivariate analysis. In our final analysis, we determined the goodness-of- fit indices for our model and examined both unadjusted & adjusted odds ratios.

We constructed a multivariable logistic-regression model for PIh, entering age, Gender, modified shock index, Shock index, Obstructive lung disease, Induction agents, Disease conditions, EPSS, and TAPSE. Receiver-operating-characteristic curves were generated from the predicted accuracy of the TAPSE model (AUC = 0.584).

## Results

We enrolled 172 patients who met the inclusion criteria, 71 (41.2%) of whom developed PIh. From Table 1, it is observed that patients with obstructive lung disease had a significantly higher risk of developing hypotension post-intubation (59.3% vs. 40.7%, *p* = 0.039). It was observed that there were differences in the incidence of PIh among patients presenting with different disease conditions where intubation was indicated in the disease setting (0 = 0.049). The incidence of PIh in sepsis and respiratory failure was 66.7% and 51%, respectively.

**Table 1:** Descriptive characteristics of patients and occurrence of PIh

Measures	Hypotension		*p*-value
	YES	NO	
(*N* = 172)	**71(41.2)**	**95(55.2)**	
Age			
< 65 years	41 (39.8)	62 (60.2)	0.632
65 years	30 (43.5)	39 (59.5)	
Gender			
Male	43 (38.7)	68(61.3)	0.361
Female	28(45.9)	33(54.1)	
Co-Morbidities			
Diabetes	22(39.3)	34(60.7)	0.712
Hypertension	34(42)	47(58)	0.861
Ischemic Heart Disease	12(54.5)	10(45.5)	0.176
Chronic Renal Disease	3(42.9)	4(57.1)	0.931
Chronic Liver Disease	3(42.9)	4(57.1)	0.931
Obstructive Lung Disease	16(59.3)	11(40.7)	0.039
Disease Conditions			
Altered Mental Status	10(35.7)	18(64.3)	0.049
Cardiac Failure	4(40)	6(60)	
Head injury with low GCS and Airway threat	5(18.5)	22(81.5)	
ICH/Stroke	8(34.8)	15(65.2)	
Respiratory Failure	26(51)	25(49)	
Sepsis	10(66.7)	5(33.3)	
Others	8(44.4)	10(55.6)	

Columns present the summary of the distribution of PIh based on demographic factors, comorbidities, and disease conditions in 172 patients, of which 71 (41.2%) had post-intubation hypotension

*P*-values derived from chi-squared tests are shown for statistical significance. *GCS* Glasgow Coma Scale, *ICH* Intracranial Haemorrhage

From Table 2, we observed that the emergency teams used mainly Etomidate, Ketamine and Propofol, and the incidence of PIh in this context was 40.7%, 42.9% and 40% of subjects, respectively (*p* = 0.980). There was one case where midazolam was used for induction, which was not included in the above data for analysis. Supplementary Table 3 shows a comparison of patient characteristics by induction agent and PIh.

**Table 2:** Medications used for induction

Medications (*n* = 171*)	Hypotension (*n*, %)		X^2^ Value	*p*-value
	Yes	No		
Etomidate	55(40.7)	80(59.3)	0.040	0.980
Ketamine	9(42.9)	12(57.1)		
Propofol	6(40)	9(60)		

This table presents a summary of medications used for induction and their correlation with PIh

*n = 1 induction with Midazolam, excluded from analysis

**Fig. 5:**
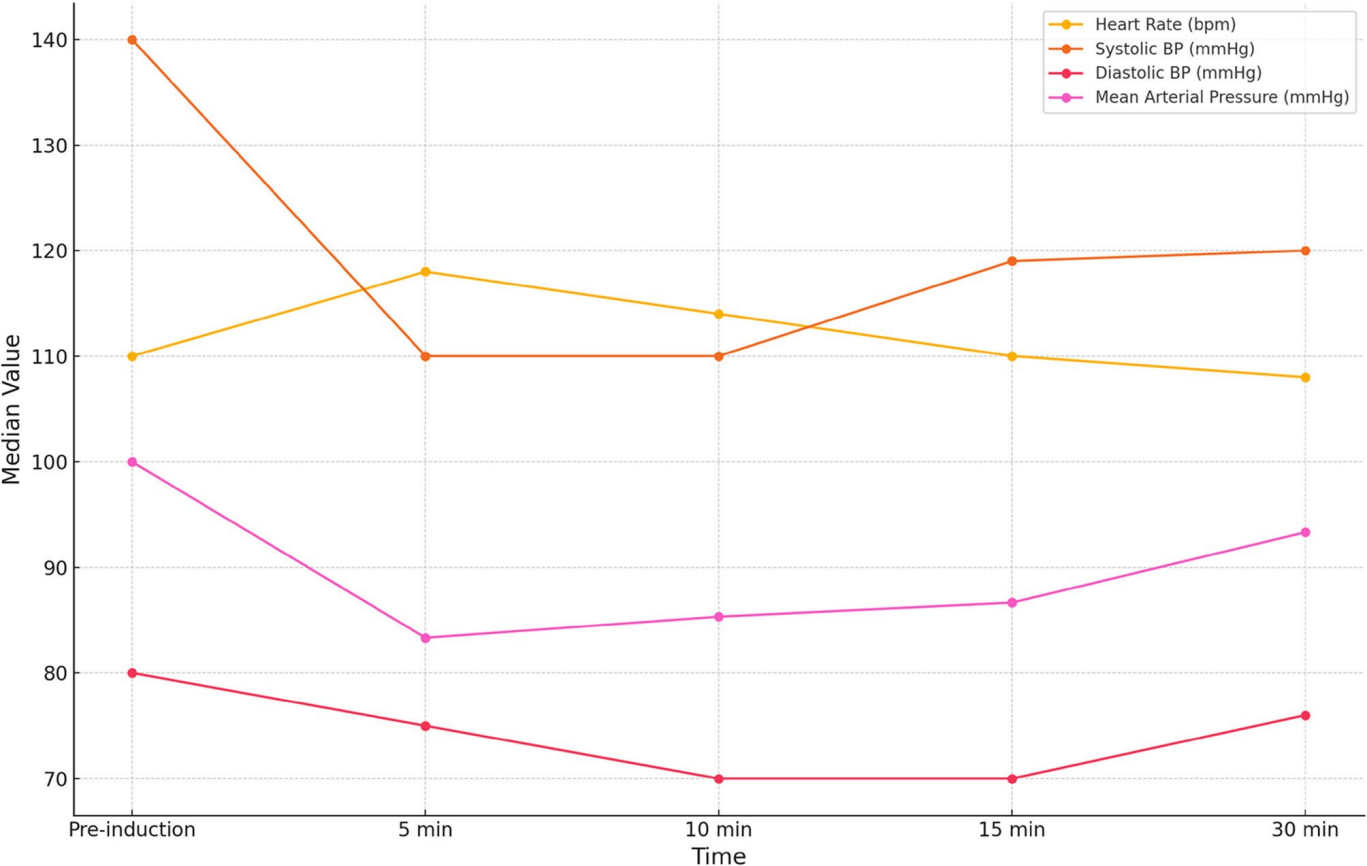
Changes in cardiovascular parameters in patients with PIh

Figure 5 shows hemodynamic changes post-intubation over the measured time intervals. The median heart rate increased by 7.27% at 5 min post-intubation (110 bpm to 118 bpm) and gradually returned to baseline at 30 min. The systolic blood pressure dropped by 21.43% at 5 min post-intubation. A similar reduction was observed at 10 min (21.43%), with a slight recovery at 30 min. A parallel trend was observed in the decrease in mean arterial pressure (16.67%) at 5 min, with a gradual recovery at 30 min. A tabulated form of the same is available in the Supplementary material as Supplementary Table 1.

Table 3 compares the pre-intubation point-of-care variables and their association with PIh. This revealed that the TAPSE values were significantly lower in patients who developed hypotension than those who did not (17.66 ± 2.45 mm vs. 18.54 ± 2.15, *p* = 0.014). It was also noted that there was no significant difference in the EPSS values and IVC Collapsibility indices between the two groups (*p* = 0.983 and *p* = 0.655, respectively).

**Table 3:** Echocardiographic parameters predicting PIh

Predictors	Hypotension		*p*-value
	YES	NO	
	Mean ± SD		
ECHO pre-induction			
EPSS Value Pre-induction	5.420 ± 1.807	5.413 ± 2.298	0.983
TAPSE Value Pre-induction	17.663 ± 2.456	18.545 ± 2.151	**0.014**
IVC Collapsibility Index Pre-induction	0.42 ± 0.200	0.43 ± 0.14	0.655

This table compares the point-of-care echocardiography parameters (EPSS and TAPSE) between patients who had PIh (*N* = 71) and those who did not (*N* = 101). The *p*-values were derived from t-tests

*EPSS* End Point Septal Separation, *TAPSE* Tricuspid Annular Plane Systolic Excursion, *IVC* Inferior vena cava, *SD* Standard Deviation

Table 4 shows a multivariate logistic regression that identified TAPSE as an independent predictor of PIh (OR = 0.81, 95% CI = 0.69–0.95, *p* = 0.009). Modified shock index approached significance in the unadjusted model in Supplementary Table 2(UOR 2.270, 95% CI = 0.951–5.418, *p* = 0.065) but was insignificant after adjustment.

**Table 4:** Multivariate logistic regression analysis of predictors of PIh

Predictor	*p*-value	Odds ratio	95% CI	
			Lower	Upper
Age	0.230	0.99	0.97	1.01
Obstructive Lung Disease	0.480	1.51	0.47	4.76
EPSS Value Pre Induction	0.910	1.01	0.87	1.18
TAPSE Value Pre Induction	0.009	0.81	0.69	0.95
Shock Index Pre Induction	0.870	1.13	0.28	4.59
Modified Shock Index Pre Induction	0.540	1.37	0.50	3.76
Gender	0.510	1.32	0.57	3.03
Disease Condition- Medical	0.800	1.13	0.44	2.87
Disease Condition- Trauma	0.970	0.98	0.38	2.49
Medications for Induction	0.420	0.71	0.29	1.76

**Fig. 6:**
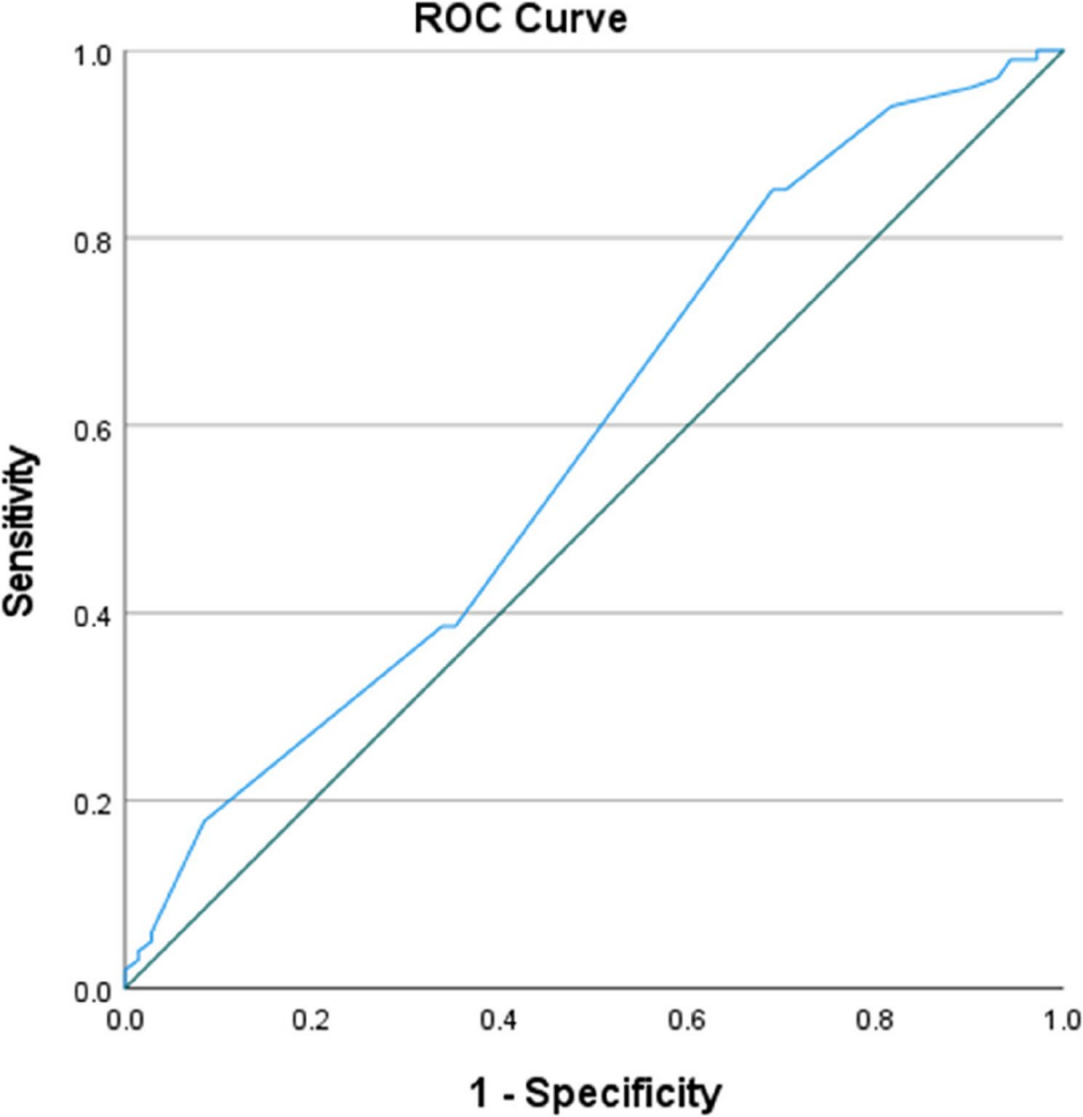
ROC model for TAPSE and post-intubation hypotension. The ROC curve demonstrates that TAPSE has moderate discriminatory performance for PIh

The predictive model performance, as shown in Fig. 6, had an AUC of 0.584 (95% CI 0.497–0.672, *p* < 0.05), indicating moderate discrimination for PIh. Using Youden’s index to select the optimal threshold, a TAPSE cut-off of 17.6 mm maximised the combined sensitivity and specificity (Table 5). At this value, sensitivity was 85.15% (identifying the majority of patients who subsequently developed hypotension), while specificity was 29.58% (correctly excluding nearly one-third of patients who remained stable). An LR + of 1.23 indicates that a TAPSE < 17.6 mm only slightly increases the odds of PIh, while an LR- of 0.48 means a TAPSE ≥ 17.6 mm cuts the odds of hypotension by roughly half, which has a modest “rule out” ability.

**Table 5:** Diagnostic accuracy of predictors for Pih

Variable	AUC	OR (95% C.I)	Cut-offs	Sensitivity (%)	Specificity (%)	PPV (%)	NPV (%)	Diagnostic accuracy (%)	LR (+)	LR (-)
TAPSE	0.584	0.061 (0.497–0.672)	17.6	85.15	29.58	63.24	58.33	53.3%	1.23	0.48

This model shows a TAPSE Cut-off of 17.6 mm, chosen by Youden’s index (maximizing sensitivity + specificity − 1)

AUC Area under curve, *OR* Odds ratio, *PPV* Positive predictive value, *NPV* Negative predictive value, *LR +* Positive likelihood ratio, *LR-* Negative predictive value

The graph summarizes changes in cardiovascular parameters in patients with Post-intubation hypotension. The data includes Heart rate, Systolic blood pressure(SBP), Diastolic blood pressure (DBP), and Mean arterial pressure (MAP) measured at five time points: pre-induction, 5, 10, 15 and 30 min post-intubation.

##  Discussion

The primary aim of our study was to identify the time sensitive clinical predictors of PIh among patients undergoing drug-assisted intubation in the Emergency Medicine Department. The TAPSE values measured pre-induction were determined to be one of the key clinical predictors among patients who had hypotension after intubation. It was observed that lower TAPSE values served as a reliable point-of-care echocardiographic marker for an Emergency Physician to predict PIh.

Our findings demonstrated that patients who were measured to have a TAPSE value of below 17.6 mm were more likely to develop PIh. This indicates the significant clinical utility of TAPSE in identifying high-risk patients who present to the emergency departments. Even though this association was found to be sensitive in detecting at-risk patients, its low specificity suggests that it should be used in conjunction with other clinical parameters rather than as a standalone rule.

In addition to this, it was also noted that the modified shock index was significant in providing information on the hemodynamic status, with higher MSI values associated with an increased occurrence of PIh.

The association between lower TAPSE values and PIh may be explained by its impact on the response to preload of the right ventricle and myocardial contractility. The right ventricle is thin-walled and is highly sensitive to increases in afterload and preload. A dysfunctional RV leads to interventricular septal bowing, leading to a reduced left ventricular preload and a compromised cardiac output due to interventricular dependence. This compromised ability, coupled with the mechanical effects of positive pressure ventilation post-intubation, could lead to acute reductions in cardiac output and hence result in hypotension [4]. 

Elevated pulmonary pressures also impair RV coronary perfusion, causing ischemia and further reduction in RV contractility, creating a vicious cycle of hemodynamic deterioration [4]. 

This also coincides with a review article by Mosier (2024) where a right ventricular dysfunction, with a TAPSE < 16 mm, amounts to a physiologically difficult airway with increased peri-intubation complications [11]. 

The findings from our study also support a previous study by Al-Saadi et al. (2022), where pre-existing moderate and severe RV dysfunction significantly increased the likelihood of post-intubation cardiac arrest and hemodynamic instability [12]. 

Both of our studies emphasize the importance of pre-intubation echocardiographic assessments, with a similar focus on TAPSE as a measure of right ventricular dysfunction in emergency care settings.

Our study also found an interesting trend in the hemodynamic parameters among the patients who experienced PIh, as evidenced in Fig. 5. The early fall in blood pressure is Likely due to the combined vasodilatory effects of induction agents, loss of sympathetic tone, and abrupt application of positive pressure ventilation, reducing venous return. The initial tachycardia response observed can be representative of a compensatory sympathetic surge in order to preserve the cardiac output. Near normalisation of the hemodynamic parameters to baseline over the next 15–30 min could be secondary to the combined effects of endogenous catecholamine response, exogenous vasopressors and a redistribution of intravascular volume due to activation of reflex pathways. This time-course data highlights that PIh is most severe in the first 5–10 min, with a partial recovery by half an hour, underlining the importance of close hemodynamic monitoring in the pre, peri and immediate post-intubation period among high-risk patients. The establishment of invasive blood pressure monitoring will be a desirable monitoring in this venture for such patients, where non-invasive blood pressure monitoring may have delays in timely identifying hypotensive events.

### Clinical implication

The observations from this study reinforce the utility of POCUS in the emergency department, specifically the importance of TAPSE. This non-invasive, crucial bedside tool for rapid assessment specifically evaluates RV systolic performance, reflecting RV dysfunction severity, providing critical insights into a patient’s right ventricular function, and guiding more informed clinical decisions. This also indicates that only eyeballing or roughly estimating the left heart function is not adequate in predicting the occurrence of hypotension after intubation, but also suggests considering the significant role the right ventricle plays in hemodynamics. Even with the decent sensitivity that this tool exhibited in the study, incorporating TAPSE into broader assessments of shock indices, clinical variables, and operator judgement will ensure that the at-risk patients are identified and adequate countermeasures to prevent PIh are taken.

A truly comprehensive pre-intubation evaluation begins with the basics—heart rate, blood pressure, and simple shock indices—but then adds a targeted, rapid POCUS (TAPSE for RV systolic function, EPSS for LV function, and IVC assessment for volume status), along with a comprehensive airway assessment. This three‐view exam takes under a minute and can be performed in parallel with IV line placement and drug preparation. Critically, the team must also review the patient’s comorbidities and drug history as these alter preload, afterload, and baseline RV mechanics. This too can be indirectly assessed by the incorporation of a focused echocardiography, which primarily focuses on the physiology of the patient and helps tailor interventions for patients at risk for PIh [13]. 

To embed this into practice, we recommend formalising a POCUS checklist that includes a preparatory phase with a dedicated cardiac probe attached to the airway cart, ensuring parallel workflows where an assistant draws up induction drugs and vasopressors while the operator acquires the three POCUS views and documents the measurements. The establishment of invasive blood pressure monitoring will be a better modality in comparison with non-invasive blood pressure monitoring, which may have delays in timely identifying hypotensive events. Tailored cardio-stable and stress-free induction plan, understanding of potential drug interactions and application of individualised ventilator settings for high-risk patients can also be incorporated. Team coordination by engaging physicians, nurses and respiratory therapists in training sessions so that probe setup, image acquisition, and documentation flow seamlessly alongside medication administration and monitor setup.

By integrating a structured POCUS protocol, backed by brief, hands-on training modules, quick image‐archiving into the electronic record, and clear multidisciplinary roles, the emergency physicians can identify hemodynamic vulnerabilities in real time, tailor induction choices, and mitigate the risk of PIh without adding significant delays to critical airway management.

### Future research direction

Future research using larger sample sizes across diverse clinical settings may consider the predictors of PIh based on multiple echocardiographic indices.

The results of this study could inform updates to emergency airway management guidelines and training programs.

Findings from this study can also inspire predictive tools for physiologically difficult airway risk scoring.

Engaging multiple disciplines like anaesthesia, critical care, respiratory therapy and nurses to adapt their checklists to include crucial echocardiographic findings and form a unified pre-intubation policy.

### Limitations

Our sample size was 172 patients in a unicentric study; however, it will be imperative to analyze larger data sets to generalize the findings across diverse pathological presentations with multicentric studies. It was beyond the scope of the observational study to obtain both pre-induction and post-intubation arterial blood gas analysis, as they were performed as per the discretion of the treating clinician. Hence, the effect of factors like pH, PaCO_2_ levels and ionic fluctuations would have been under-recognized despite the potential of their influence due to their complex interactions with implications in cardiovascular functions.

Although we captured regular antihypertensive and diuretic use, we did not adjust for these chronic medications in our primary analysis. It did not yield a significant correlation due to variability and the overlapping nature of medications used in patients. These factors can influence preload and afterload and, therefore, remain potential unmeasured confounders in our study.

## Conclusion

Our study identifies several vital predictors of PIh in patients undergoing drug-assisted intubation in the emergency medicine department. An emergent assessment of the right ventricular function was performed by measuring the tricuspid annular plane systolic excursion (TAPSE) values, which suggested that lower values were associated with an increasing risk of PIh. This highlights the value of time-sensitive and time-restricted point-of-care echocardiography in the emergency setting, which includes assessment of the right ventricular function as a predictor for PIH in high-risk patients.

## Supplementary Information

Supplementary Material 1

## Data Availability

No datasets were generated or analysed during the current study.

## Abbreviations

PIh: Post-intubation hypotension
POCUS: Point-of-care ultrasonography
TAPSE: Tricuspid Annular Plane Systolic Excursion
EPSS: End Point Septal Separation
SBP: Systolic blood pressure
MAP: Mean arterial pressure
SPSS: Statistical package for social sciences
ROC: Receiver operating characteristics
AUC: Area under the curve
MSI: Modified shock index
RV: Right Ventricle
LV: Left Ventricle
ABG: Arterial Blood gas
PaCO2: Partial pressure of Carbon dioxide
