# Supplementary material for: Right ventricular dysfunction: a key predictor of post-intubation hypotension in the emergency department
Source: Int J Emerg Med. 2025 Sep 30;18:183. doi: 10.1186/s12245-025-00987-0 (PMC12487513; doi:10.1186/s12245-025-00987-0)
Supplement: Supplementary file 1 — Supplementary Material 1 [file 12245_2025_987_MOESM1_ESM.docx]

**Supplementary Table 1: Drop in Blood Pressures Among Group that experienced PIh**

| **Hypotension – Yes (N=71)**  **Time** | | **Median Value** | **Median Change** | **Percentage Change** |
| --- | --- | --- | --- | --- |
| Heart Rate | **Pre-induction** | **110** |  |  |
|  | 5 Mins Post-intubation | 118 | -1.6 | 7.27% |
|  | 10 Mins Post-intubation | 114 | 0.00 | 3.64% |
|  | 15 Mins Post-intubation | 110 | 0.00 | 0 |
|  | 30 Mins Post-intubation | 108 | -0.95 | -1.82% |
| Systolic Blood Pressure | **Pre-induction** | **140** |  |  |
|  | 5 Mins Post-intubation | 110 | 11.17 | -21.43% |
|  | 10 Mins Post-intubation | 110 | 16.92 | -21.43% |
|  | 15 Mins Post-intubation | 119 | 9.09 | -15% |
|  | 30 Mins Post-intubation | 120 | 10.19 | -14.29% |
| Diastolic Blood Pressure | **Pre-induction** | **80** |  |  |
|  | 5 Mins Post-intubation | 75 | 3.75 | -6.25% |
|  | 10 Mins Post-intubation | 70 | 10.00 | -12.50% |
|  | 15 Mins Post-intubation | 70 | 6.70 | -12.50% |
|  | 30 Mins Post-intubation | 76 | 0.00 | -5.00% |
| Mean Arterial Pressure | **Pre-induction** | **100** |  |  |
|  | 5 Mins Post-intubation | 83.33 | 8.54 | -16.67% |
|  | 10 Mins Post-intubation | 85.33 | 12.07 | -14.67% |
|  | 15 Mins Post-intubation | 86.66 | 9.09 | -13.34% |
|  | 30 Mins Post-intubation | 93.33 | 5.37 | -6.67% |

This table presents the hemodynamic changes in patients (N=71) with PIh. The data includes Heart rate, Systolic blood pressure(SBP), Diastolic blood pressure (DBP), and Mean arterial pressure (MAP) measured at five time points: pre-induction, 5, 10, 15 and 30 minutes post-intubation.

**Supplementary Table 2: Bivariate Logistic regression table**

| Variable | Hypotension | | Unadjusted  OR | 95% CI | p-value |
| --- | --- | --- | --- | --- | --- |
|  | **Yes** | **No** |  |  |  |
| Age | 59.56$\pm$15.02 | 57.25$\pm$16.99 | 1.009 | 0.990 – 1.028 | 0.356 |
| Obstructive Lung Disease | 16(59.3) | 11(40.7) | 0.420 | 0.182- 0.971 | **0.042** |
| Indications for Intubation | 71 (41.3%) | 101 (58.7%) | 0.761 | 0.392 – 1.480 | 0.421 |
| TAPSE Value Pre-induction | 17.66 $\pm$ 2.45 | 18.54 $\pm$2.15 | 0.822 | 0.695- 0.972 | **0.022** |
| Modified Shock Index Pre-induction | 1.12 $\pm$ 0.35 | 1.02 $\pm$0.35 | 2.270 | 0.951 – 5.418 | 0.065 |

Patients with underlying obstructive lung disease had 58% lower odds of developing PIh compared to those without (OR 0.420; p=0.042). Higher TAPSE- better RV systolic function was associated with an 18% reduction in odds of PIh (OR 0.822; p=0.022). Higher pre-intubation MSI trended toward increased PIh risk (OR 2.270; p=0.065) but did not reach statistical significance.

**Supplementary Table 3: Induction agents and Dosage**

| **Medications for Induction** | **Variables** | **Hypotension**  **(Mean** $\boldsymbol{\pm SD}$**) (N)** | | **p-value** |
| --- | --- | --- | --- | --- |
|  |  | **Yes** | **No** |  |
| **Etomidate** | Body Weight | 62.18$\pm$10.35  (55) | 64.95 $\pm$9.17  (80) | 0.032 |
|  | Dose of Induction Agent | 13$\pm$3.52  (55) | 13.05$\pm$3.88  (80) | 0.903 |
|  | Age | 59.93$\pm$15.03  (55) | 54.74$\pm$17.48  (80) | 0.152 |
| **Ketamine** | Body Weight | 60.89 $\pm$12.66  (9) | 58.08 $\pm$6.48  (12) | 0.002 |
|  | Dose of Induction Agent | 60 $\pm$19.36  (9) | 73.33$\pm$24.24  (12) | 0.142 |
|  | Age | 57.78 $\pm$13.75  (9) | 64.75$\pm$11.93  (12) | 0.735 |
| **Midazolam** | Body Weight | 80  (1) |  | NA |
|  | Dose of Induction Agent | 8  (1) |  |  |
|  | Age | 63  (1) |  |  |
| **Propofol** | Body Weight | 60.83$\pm$11.19  (6) | 68.78$\pm$9.48  (9) | 0.461 |
|  | Dose of Induction Agent | 63.33$\pm$23.38  (6) | 64.44$\pm$7.26  (9) | 0.054 |
|  | Age | 58.33$\pm$20.10  (6) | 69.56$\pm$8.80  (9) | 0.123 |

This table comprises of comparison of patient characteristics by induction agent and post-intubation hypotension. Patients who developed PIh after etomidate induction were slightly lighter (62.2 kg vs. 64.9 kg; p=0.032), though induction doses and ages did not differ significantly. Among ketamine users, those with PIh weighed more on average (60.9 kg vs. 58.1 kg; p=0.002), but induction dose and age differences were non-significant. No significant differences in weight, dose, or age between hypotensive and non-hypotensive groups where Propofol was used as an induction agent. Single midazolam case precludes statistical comparison.

These findings suggest that, for etomidate and ketamine, small but statistically significant differences in patient body weight may correlate with PIh risk, while induction dose and age were not driving factors. No such patterns were observed with propofol. This supports close attention to baseline anthropometrics when selecting induction agents
